# Integrated analysis reveals distinct molecular, clinical, and immunological features of *B7‐H3* in acute myeloid leukemia

**DOI:** 10.1002/cam4.4284

**Published:** 2021-09-25

**Authors:** Ling‐yi Zhang, Ye Jin, Pei‐hui Xia, Jiang Lin, Ji‐chun Ma, Ting Li, Zi‐qi Liu, He‐lin Xiang, Chen Cheng, Zi‐jun Xu, Hong Zhou, Jun Qian

**Affiliations:** ^1^ Laboratory Center Affiliated People’s Hospital of Jiangsu University Jiangsu China; ^2^ Zhenjiang Clinical Research Center of Hematology Jiangsu China; ^3^ Department of Hematology Affiliated People’s Hospital of Jiangsu University Jiangsu China; ^4^ School of Medical Science and Laboratory Medicine Jiangsu University Jiangsu China

**Keywords:** acute myeloid leukemia, *B7‐H3*, immune checkpoint, prognosis

## Abstract

The role of *B7*‐*H3* in acute myeloid leukemia (AML) is not fully understood. Two previous studies investigating its expression and significances in AML are partially different. In this study, we aimed to systematically characterize the genomic and immune landscape in AML patients with altered *B7*‐*H3* expression using multi‐omics data in the public domain. We found significantly increased *B7*‐*H3* expression in AML compared to either other hematological malignancies or healthy controls. Clinically, high *B7*‐*H3* expression was associated with old age, *TP53* mutations, wild‐type *WT1* and *CEBPA*, and the M3 and M5 FAB subtypes. Moreover, we observed that increased *B7*‐*H3* expression correlated significantly with a poor outcome of AML patients in four independent datasets. Gene set enrichment analysis (GSEA) revealed the enrichment of the “EMT” oncogenic gene signatures in high *B7*‐*H3* expressers. Further investigation suggested that *B7*‐*H3* was more likely to be associated with immune‐suppressive cells (macrophages, neutrophils, dendritic cells, and Th17 cells). *B7*‐*H3* was also positively associated with a number of checkpoint genes, such as *VISTA* (*B7*‐*H5*), *CD80* (*B7*‐*1*), *CD86* (*B7*‐*2*), and *CD70*. In summary, we uncovered distinct genomic and immunologic features associated with *B7*‐*H3* expression in AML. This may lead to a better understanding of the molecular mechanisms underlying *B7*‐*H3* dysregulation in AML and to the development of novel therapeutic strategies.

## Graphical abstract

In this paper, we found significantly increased B7‐H3 expression in AML compared to either other hematological malignancies or healthy controls. Importantly, we observed that increased B7‐H3 expression correlated significantly with a poor outcome of AML patients in four independent datasets. We also uncovered distinct genomic and immunologic features associated with B7‐H3 expression in AML.

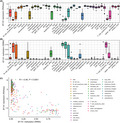

## 1 INTRODUCTION

Acute myeloid leukemia (AML) is a group of heterogeneous diseases characterized by diverse cytogenetic aberrations and variable responses to therapy. Identification of essential molecular abnormalities underlying leukemogenesis could help facilitate better therapeutic decisions.

Recent progress in cancer immunology has contributed to the compelling success of immune checkpoint blockade (ICB) therapy.^1^ However, sustainable responses to ICB have been observed in only a minority of patients, and the impressive success of ICB in a subset of solid tumors has yet to be translated fully to hematological malignancies.^2^, ^3^, ^4^ Hematological malignancies, especially AML, are characterized by a distinct immune microenvironment: they have originated from bone marrow (BM) where most immune cells develop and reside, thus making them highly immunosuppressive.^5^ For example, we recently reported that M2 macrophages, cells that exhibit pro‐tumor and immunosuppressive phenotypes, are preferentially enriched in AML compared with normal BM.^6^ We have also identified the M2 marker *CD206* as a strong predictor of adverse outcomes in AML patients. Furthermore, negative regulatory immune checkpoint molecules, such as *PD*‐*L1*, *CTLA*‐*4*, *LAG*‐*3*, *TIM*‐*3*, and *IDO1*, have all been demonstrated to be up‐regulated in AML and some of them may become promising therapeutic targets.^7^ Therefore, more efforts are needed to understand the roles of checkpoint genes in AML.

*B7*‐*H3*, also known as *CD276*, is a relatively new immune checkpoint molecule belonging to the B7‐CD28 superfamily.^8^ It was found to play both co‐stimulatory and co‐inhibitory roles in T‐cell‐mediated immune responses,^9^ with broad expression at the mRNA level in most tissues, but with limited expression at the protein level in some immune cells.^10^, ^11^ Also, the expression patterns and clinical significances of *B7*‐*H3* have been extensively studied in a wide range of solid tumors, such as lung, prostate, ovarian, pancreatic, gastric, and colorectal cancers.^12^, ^13^, ^14^, ^15^, ^16^, ^17^ In hematological malignancies, however, very few such studies have yet been performed. Although increased expression of *B7*‐*H3* in AML has been reported in two previous studies,^18^, ^19^ their analyses were restricted to relatively small sample sizes and, to date, the genetic and transcriptional features underlying *B7*‐*H3* alteration has not been thoroughly investigated. Moreover, it will also be interesting to examine the relationship between *B7*‐*H3* and tumor‐infiltrating leukocytes (TILs) as well as other checkpoint genes. Therefore, in this study, drawing on rich multi‐omics data in the public domain and state‐of‐the‐art algorithms to quantify TILs (CIBERSORT and ssGSEA),^20^, ^21^ we aimed to validate *B7*‐*H3* expression and its prognostic value in AML with an enlarged sample size and to systematically characterize the genomic and immune landscape in AML patients with altered *B7*‐*H3* expression.

## 2 MATERIALS AND METHODS

### 2.1 Patients and database

Transcriptome‐wide gene expression data (RNA‐seq and RNA microarray) and DNA methylation profiling data (Reduced Representation Bisulfite Sequencing; RRBS) for over 1000 cancer cell lines were accessed from Cancer Cell Line Encyclopedia (CCLE) (https://www.broadinstitute.org/ccle). RNA‐seq data of 64 cell lines from The Human Protein Atlas (HPA) (https://www.proteinatlas.org/) were utilized to validate *B7*‐*H3* expression patterns in cell lines. Molecular data for the TCGA LAML project, including mRNA expression (RNA‐seq and RNA microarray), copy number, mutation data, and clinical information, were downloaded from TCGA data portal (https://gdc.nci.nih.gov). The study was approved by the Washington University Human Studies Committee, and written informed consents were obtained from patients. In addition, we used six microarray data obtained from Gene Expression Omnibus (GEO) (http://www.ncbi.nlm.nih.gov/geo), under accession numbers GSE13159, GSE63270, GSE30029, GSE10358, GSE12417 (U133plus2), and GSE71014. As for the GEO datasets, all informed consents were obtained from respective cohorts. The former three datasets contain both healthy and AML samples. The latter three for which survival data were available were used to analyze the association between *B7*‐*H3* and patient outcome. For a more detailed description of these datasets, please refer to our previous publications.^6^, ^22^ In case of multiple probes per gene, *B7*‐*H3* expression was determined using a probe set with the highest mean expression.

### 2.2 Estimation of immune cell fractions

The relative abundances of 22 immune cell populations were estimated using CIBERSORT as previously described.^6^, ^20^ As CIBERSORT may not be suitable for the use of the RNA‐seq data,^23^ this algorithm was exclusively applied to the TCGA microarray, GSE10358, and GSE13159 datasets. To give a more robust estimation of immune infiltration, we further collected four sets of immune gene signatures published by Angelova et al,^24^ Bindea et al,^25^ Charoentong et al,^26^ and Senbabaoglu et al.^27^ The single sample Gene Set Enrichment Analysis (ssGSEA) method,^21^ as implemented in the R Bioconductor package gsva,^28^ was then introduced to quantify enrichment scores of respective cell populations in each collection. ssGSEA signature scores between patients with high and low *B7*‐*H3* expression were compared using the limma package.^29^ The p values were adjusted for multiple testing using the Benjamini–Hochberg procedure. Furthermore, 20 immune cell types from the Charoentong et al collection were divided into cell types executing anti‐tumor immunity (Activated CD4 T cell, Activated CD8 T cell, Central memory CD4 T cell, Central memory CD8 T cell, Effector memory CD4 T cell, Effector memory CD8 T cell, Type 1 T helper cell, Type 17 T helper cell, Activated dendritic cell, CD56bright natural killer cell, Natural killer cell, and Natural killer T cell) and cell types executing pro‐tumor, immune suppressive functions (Regulatory T cell, Type 2 T helper cell, CD56dim natural killer cell, Immature dendritic cell, Macrophage, MDSC, Neutrophil, and Plasmacytoid dendritic cell), according to Jia et al.^30^ The ssGSEA score of the respective group were summarized to represent the abundances of these two categories.

### 2.3 Statistical analysis and bioinformatics

Overall survival (OS) was defined as the time from the date of diagnosis to death due to any cause. Event‐free survival (EFS) was defined as the time from diagnosis to disease relapse, progression, or death due to any cause. Survival analysis was conducted in R using the “survival” library. The optimal cutoff points of *B7*‐*H3* expression were determined by maximally selected rank statistics (maxstat) implemented in the “survminer” R package. Survival probabilities of patient groups were estimated by the Kaplan–Meier method and compared with log‐rank test.

We used Wilcoxon rank‐sum test to compare differences between two groups, or one‐way ANOVA for more than two groups, followed by multiple pairwise‐comparison using Tukey’s test. To determine statistical significance between the means of each patient group from GSE13159, we used one‐way ANOVA, since the largest standard deviation (AML, 0.94) is less than double the smallest standard deviation (normal, 0.65). Correlation between two continuous variables was computed using Spearman's rank correlation method. Differential gene expression analysis for TCGA RNA‐seq data was calculated using the raw read counts with the R/Biocondcutor package “edgeR”.^31^ Gene set enrichment analysis (GSEA) was performed using GSEA v4.0 software (http://www.broad.mit.edu/gsea), with oncogenic and hallmark gene sets obtained from the Molecular Signatures Database (MSigDB). The gene sets were considered to be significantly enriched at a false discovery rate <0.25 and normalized *p*‐value <0.05. We applied STRING (Search Tool for the Retrieval of Interacting Genes) (http://string.embl.de/) to construct a protein–protein interaction (PPI) network of the differentially expressed genes (DEGs). We chose a confidence score >0.4 as the judgment criterion. Cytoscape visualization software (version 3.6.1) was used to present the *B7*‐*H3*‐related sub‐network.

The genetic alterations of *B7*‐*H3* in pan‐cancers, including somatic mutations, amplification, and deep deletion were assessed through the cbioportal for Cancer Genomics (http://www.cbioportal.org). Differentially mutated genes between two cohorts were identified with the mafCompare function in “Maftools” package.^32^ To detect copy number alterations (deletions and amplications) in AML patients, we analyzed filtered segmented copy number data (Affymetrix SNP 6.0 platform) using the GISTIC 2.0 algorithm.^33^ Visualization was performed using the following R packages: “ggplot2”, “ggsci”, “ggpubr”, “pheatmap”, “PairViz” (for network of multiple pairwise‐comparison analysis), “circlize” (for circos plot), and “Maftools” (for forest plot and co‐onco plot). All statistical tests were two‐sided, and a p‐value less than 0.5 was considered to indicate statistical significance.

## 3 RESULTS

### 3.1 B7‐H3 expression in human cell lines

**FIGURE 1:**
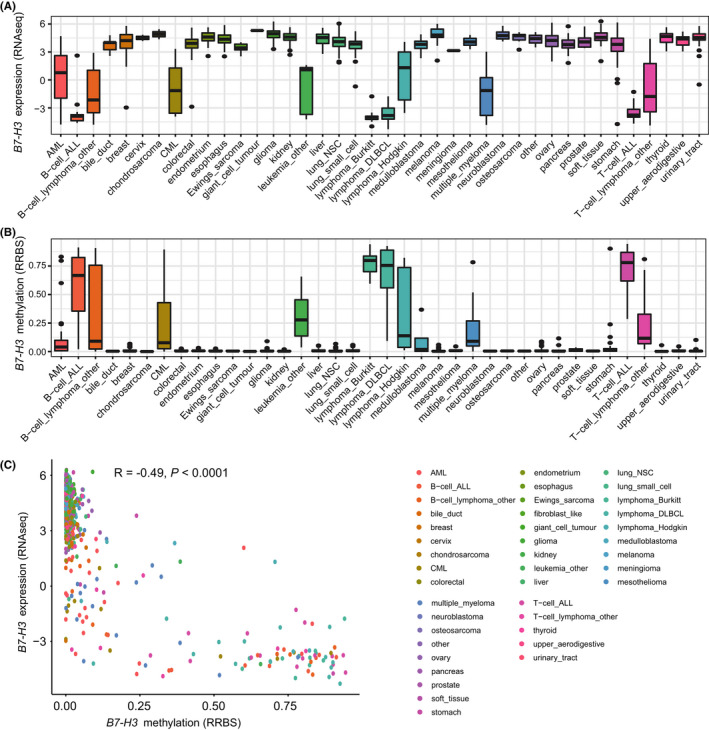
The expression and methylation levels of *B7*‐*H3* in cancer cell lines. (A) *B7*‐*H3* mRNA expression levels (RNA‐seq data) across cancer cell lines from the Cancer Cell Line Encyclopedia (CCLE) (https://www.broadinstitute.org/ccle). (B) *B7*‐*H3* DNA methylation levels across cancer cell lines from the Cancer Cell Line Encyclopedia (CCLE) (https://www.broadinstitute.org/ccle). (C) Correlation between *B7*‐*H3* mRNA expression and DNA methylation levels in the CCLE datasets. (R, Spearman correlation coefficient)

To examine the expression patterns of *B7*‐*H3* in cancers at a large scale, we first exploited the RNA‐sequencing data of over 1,000 cell lines from the Cancer Cell Line Encyclopedia (CCLE) (https://www.broadinstitute.org/ccle). We found that *B7*‐*H3* is robustly expressed in cell lines of solid tumors and glioma. For hematologic malignancies, the expression of *B7*‐*H3* is relatively low and more variable, with increased expression in AML compared to other myeloid malignancies like CML and the lymphoid malignancies (Figure 1A). A similar expression pattern was observed in the RNA microarray data from CCLE (Figure S1A). These results were confirmed by analyzing the RNA‐seq data of 64 cell lines from The Human Protein Atlas (HPA) (https://www.proteinatlas.org/) (Figure S1B). Using CCLE, we then examined the DNA methylation status (Reduced Representation Bisulfite Sequencing: RRBS) of *B7*‐*H3* across the cancer cell lines. Interestingly, *B7*‐*H3* was found to be unmethylated in nearly all solid tumor cell lines. Aberrant methylation of *B7*‐*H3* was detected in lymphomas and lymphoid malignancies, whereas AML showed the lowest median methylation level in all hematologic malignancies (Figure 1B). Further analysis revealed that *B7*‐*H3* expression and methylation levels were significantly negatively correlated (Figure 1C). This negative correlation was also seen when analyzing the TCGA dataset (Figure S2). This indicated that *B7*‐*H3* expression was potentially regulated by DNA methylation.

### 3.2 Gene alterations of B7‐H3 in pan‐cancers

Next, we surveyed the genetic alterations of *B7*‐*H3* in TCGA pan‐cancer datasets using the cbioportal for Cancer Genomics (http://www.cbioportal.org). The frequencies of genetic alterations regarding *B7*‐*H3*, including mutations, amplifications, and deletions, are shown in Figure S3A. We found that the overall genetic alteration rate of *B7*‐*H3* in cancers appeared to be very low: it was altered in 130 of 10950 pan‐cancer patients (1.2%, Figure S3B). Kidney chromophobe demonstrated the highest frequency of *B7*‐*H3* mutation (3.08%), followed by skin cutaneous melanoma (2.93%) and uterine corpus endometrial carcinoma (2.84%). For copy number variations (CNVs), amplifications were more commonly seen. For example, amplifications of *B7*‐*H3* were the only genetic events in mesothelioma (3.45%) and uterine carcinosarcoma (1.75%). In AML, however, no genetic alterations of *B7*‐*H3* were existed, suggesting DNA methylation might be the only reason for abnormal *B7*‐*H3* expression in AML (Figure S3A).

### 3.3 B7‐H3 expression is elevated in AML patients

**FIGURE 2:**
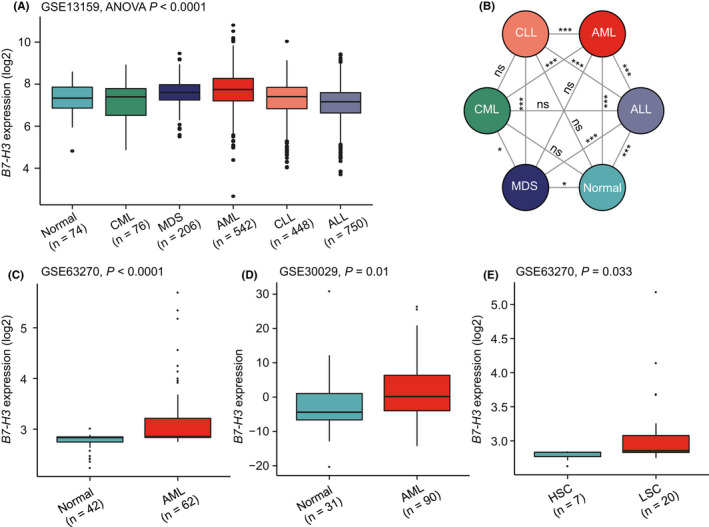
The expression of *B7*‐*H3* in primary AML samples and normal controls. (A) Box plots showing the expression of *B7*‐*H3* in five major hematological malignancies (CML, MDS, AML, CLL, and ALL) and healthy controls, using the MILE dataset (GSE13159, *n* = 2096). (B) Network diagram illustrating the p‐values of multiple pairwise‐comparison analysis for groups described in (A), using the “PairViz” package. (C, D, and E) Box plots showing *B7*‐*H3* expression levels in normal controls and AML in the GSE63270 (C) and GSE30029 (D) datasets, and in hematopoietic stem cells (HSCs) and leukemia stem cells (LSCs) from the GSE63270 dataset (E). Except for GSE30029 (D), where the original data downloaded from GEO were used for visualization, data of GSE13159 and GSE63270 (A, C, and E) were log2 transformed for boxplot presentation

We next examined the expression of *B7*‐*H3* in primary AML samples from patients. By analyzing the MILE dataset (GSE13159, *n* = 2096), we found significant differences in *B7*‐*H3* expression across five major hematological malignancies (CML, MDS, AML, CLL, and ALL) and healthy controls (ANOVA, *p* < 0.0001), with the highest level observed in AML (Figure 2A). The means and standard deviations of *B7*‐*H3* expression in each patient group were as follows: normal = 7.31 ± 0.65, CML = 7.17 ± 0.93, MDS = 7.57 ± 0.66, AML = 7.71 ± 0.94, CLL = 7.22 ± 0.9, and ALL = 7.08 ± 0.85. Patients with myeloid malignancies had higher *B7*‐*H3* expression than that with lymphoid malignancies (Figure 2A), which agreed favorably with previous observations in cell lines. Of the pairwise between‐group comparisons, there were significant differences between AML and other four groups (*p* < 0.0001) and a trend toward higher *B7*‐*H3* expression as compared with MDS (*p* = 0.066) (Figure 2B). Importantly, the finding that *B7*‐*H3* was more highly expressed in AML than normal controls was separately confirmed in two independent datasets (GSE63270, *n* = 104; GSE30029, *n* = 121) (Figure 2C,D). The increased expression of *B7*‐*H3* was also seen in leukemia stem cells (LSCs) compared with hematopoietic stem cells (HSCs) (Figure 2E).

### 3.4 B7‐H3 expression correlates with distinct genomic alterations in AML

**FIGURE 3:**
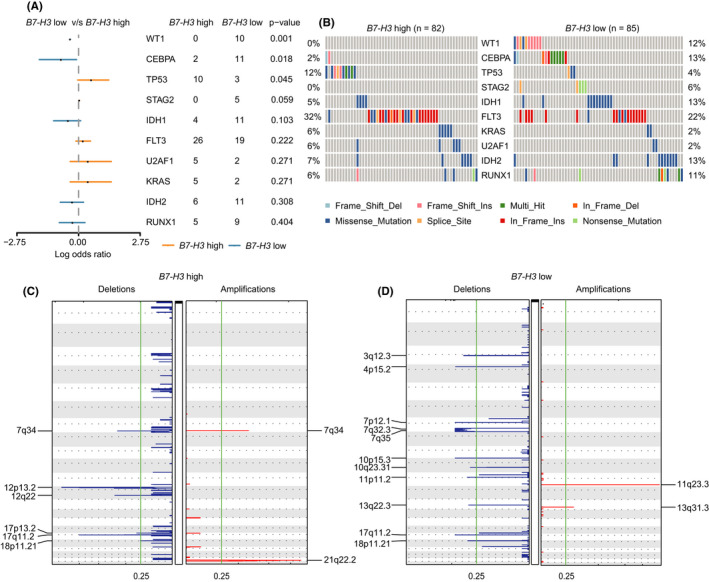
*B7*‐*H3* expression correlates with distinct genomic alterations in AML. (A) Forest plot showing the comparison of mutational profiles between patients with high and low *B7*‐*H3* expression in the TCGA dataset. (B) Co‐onco plots showing the comparison of mutational profiles between patients with high and low *B7*‐*H3* expression in the TCGA dataset. (C,D) GISTIC analyses identified recurrent copy number alterations in AML patients with high (C) and low (D) B7‐H3 expression

To determine whether *B7*‐*H3* expression correlated with distinct mutational events characterized for AML. Using curated mutational data from TCGA, we compared mutation profiles between patients with high and low *B7*‐*H3* expression (as determined by the median expression value) to detect differentially mutated genes. The results were visualized as forestplot and oncoplot (Figure 3A,B). It was found that high *B7*‐*H3* expressers were more likely to carry *TP53* mutations than low *B7*‐*H3* expressers (12% vs. 4%, *p* = 0.045). Patients with low *B7*‐*H3* expression had a higher incidence of *WT1* (12% vs. 0%, *p* = 0.001) and *CEBPA* mutations (13% vs. 2%, *p* = 0.018) than patients with high *B7*‐*H3* expression.

Regarding other clinical characteristics, patients with high *B7*‐*H3* expression tend to be older than those with low *B7*‐*H3* expression (median, 61 years versus 55 years, *p* = 0.014). *B7*‐*H3* was more highly expressed in the M3 and M5 FAB subtypes (Figure S4), consistent with previous observations.^19^ No differences were found between these two groups in terms of sex, white blood cell (WBC) count, and cytogenetic risk groups (data not shown).

Next, we performed GISTIC analyses of TCGA copy number data to identify copy number alterations in two patient groups. Overall, there was significantly fewer amplification than deletion events in both groups (Figure 3C,D). In samples with high *B7*‐*H3* expression, recurrent alterations included the deletion of short arm of chromosome 12 (12p), which contains tumor suppressors such as *CDKN1B*, *ETV6*, *DUSP16*, and *miR*‐*613*. We also identified cytokine receptor genes (*PLXNC1* and *NR2C1*) being deleted in high *B7*‐*H3* expressers. Frequently amplified genomic regions included the oncogenic driver *ERG*, whose increased expression often predicts poor outcomes in AML.^34^ For low *B7*‐*H3* expressers, significantly altered regions contain quite a few non‐coding RNAs (miRNAs and lncRNAs). One alteration regards the microdeletions on 17q (17q11.2), a region where the famous tumor suppressor gene NF1 resides.^35^

### 3.5 Distinct gene‐expression signatures associated with B7‐H3 expression in AML

**FIGURE 4:**
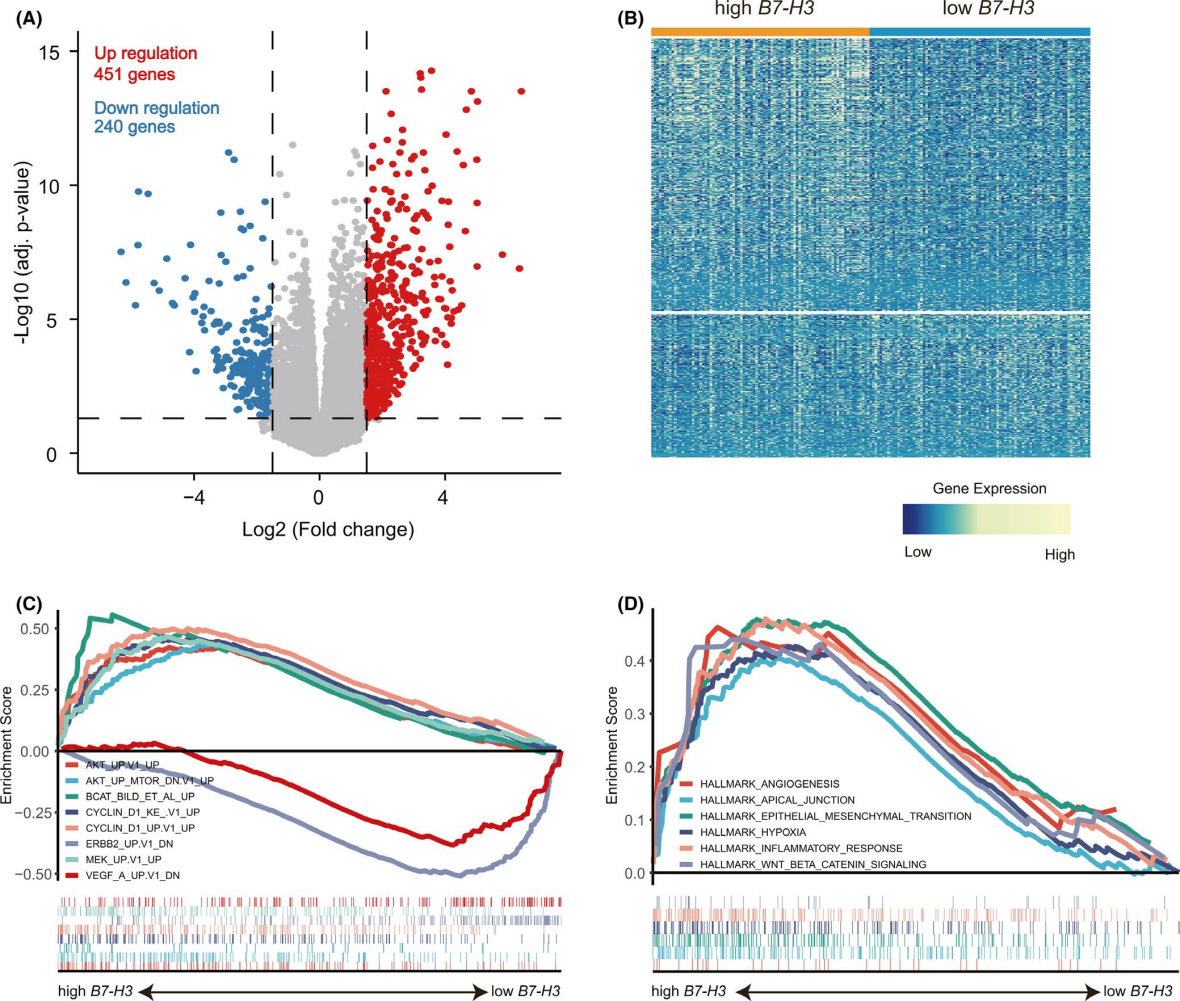
Distinct gene‐expression signatures associated with B7‐H3 expression in AML. (A,B) Volcano plot (A) and heatmap (B) showing gene expression differences between patients with low and high *B7*‐*H3* expression. Significantly down‐regulated genes (blue points) and up‐regulated genes (red points) in patients with low *B7*‐*H3* expression are indicated (FDR <0.05, |log2 FC| >1.5). (C,D) Gene set enrichment analysis (GSEA) of patients with low and high *B7*‐*H3* expression, with oncogenic (C) and hallmark (D) gene sets obtained from the Molecular Signatures Database (MSigDB)

To gain more biological insight in AML characterized by high *B7*‐*H3* expression, we performed differential gene expression using the TCGA RNAseq dataset. This analysis revealed 451 up‐regulated and 240 down‐regulated genes between patients with high and low *B7*‐*H3* expression (FDR <0.05, |log2 FC| >1.5; Figure 4A,B; Data S1). Many of the genes up‐regulated in high *B7*‐*H3* patients were, as expected, implicated in immune response. Among them were cytokines (i.e., *ADM2*, *AGT*, *BMP2*, and *CD70*), cytokine receptors (i.e., *IL17RE*, *IL1R1*, *IL1R2*, and *IL22RA1*), and chemokines (*CCL2*, *CCL19*, *CCL20*, and *CCL22*). Notably, two expression markers of the M2 macrophages‐*CD163* and *CD204*‐were found to be significantly associated with *B7*‐*H3* expression. Also up‐regulated were some oncogenes, such as *FGF10*, *MAFB*, *MRAS*, *PRDM14*, *TWIST1*, and *MMP7*. Specifically, *TWIST1* and *MMP7* are known to be involved in epithelial‐mesenchymal transition (EMT), a key process in cancer progression. In contrast, two members of the protocadherin tumor suppressor family‐*PCDH9* and *PCDH10*‐were down‐regulated in high *B7*‐*H3* expressers. We then performed a gene set enrichment analysis (GSEA) by comparing transcriptomes between cases with high and low *B7*‐*H3* expression. We found *B7*‐*H3* was positively associated with gene signatures that are up‐regulated upon overexpression of certain oncogenes, including *AKT1*, *CTNNB1*, *Cyclin D1*, and *MEK* (Figure 4C). Genes down‐regulated in cells overexpressing ligand‐activatable oncoprotein *ERBB2* and in cells treated with the angiogenic factor *VEGFA* were, on the contrary, negatively associated with *B7*‐*H3* (Figure 4C). In the high‐*B7*‐*H3* group, several cancer hallmarks including angiogenesis, apical junction, EMT, hypoxia, inflammatory response, and Wnt/beta‐catenin signaling, were significantly enriched in comparison with the low‐*B7*‐*H3* group (Figure 4D). No significant“hallmark” gene sets/hallmark signatures were enriched in the low‐*B7*‐*H3* group (FDR >0.25).

### 3.6 PPI analysis of B7‐H3 related DEGs

**FIGURE 5:**
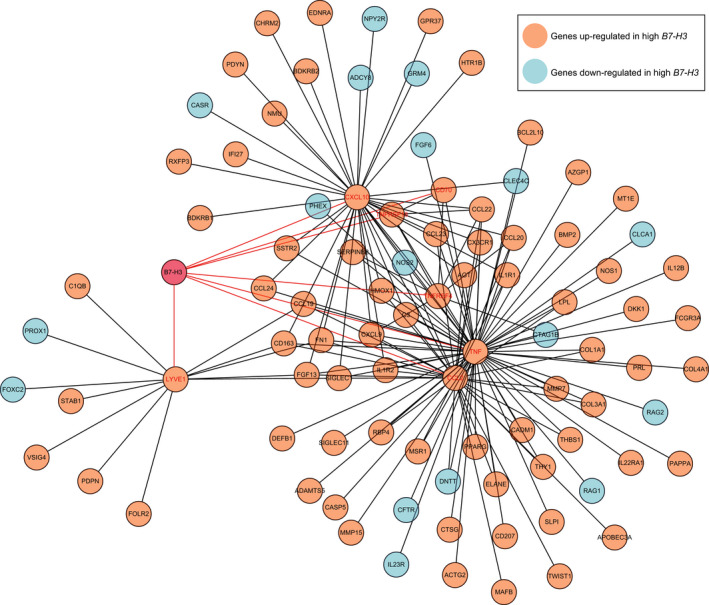
Cytoscape analysis of *B7*‐*H3*‐related sub‐network using PPI information obtained from STRING database (http://stringdb.org/). Orange represents genes up‐regulated in high *B7*‐*H3* expressers, while blue represents genes down‐regulated in high *B7*‐*H3* expressers. Genes directly interact with *B7*‐*H3* were marked in red

We then conducted a PPI network analysis of the 691 DEGs to explore the potential interactions among them. Choosing a median confidence score (0.4), 648 nodes and 1983 edges were obtained in the final network (Figure S5). In this PPI network, we found seven genes that are directly correlated with *B7*‐*H3* (*CD70*, *CXCL10*, *CCL2*, *LYVE1*, *TNFRSF18*, *TNFRSF4*, and *TNF*) (Figure 5). Notably, all seven genes were positively correlated with *B7*‐*H3* and were involved in immune response regulation. The genes directly interact with the seven genes were also shown in the network (Figure 5).

### 3.7 B7‐H3 expression is associated with immune‐suppressive cell populations in AML

**FIGURE 6:**
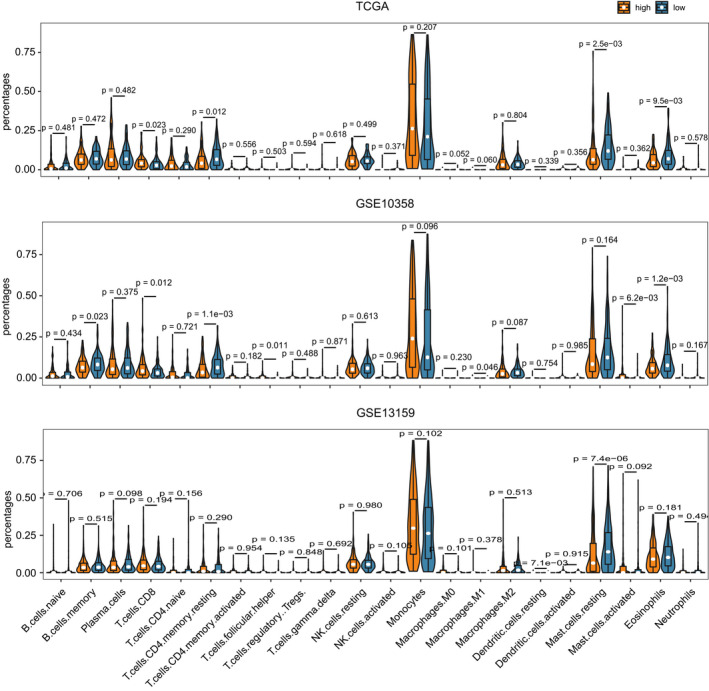
The relation between *B7*‐*H3* expression and immune cell infiltration. Violin plot showing the differences of immune cell fractions between patients with low and high *B7*‐*H3* expression. The overall immune cell compositions were estimated by CIBERSORT across three datasets (TCGA microarray, GSE10358, and GSE13159)

Since *B7*‐*H3* has classically been implicated in immune regulation, we decided to comprehensively evaluate its relation with immune cell infiltration. The overall immune cell compositions were estimated by CIBERSORT across three datasets (TCGA microarray, GSE10358, and GSE13159), for samples with high and low *B7*‐*H3* expression, respectively (Figure S6). We then compared the inferred relative fractions of 22 subpopulations between the high‐*B7*‐*H3* and low‐*B7*‐*H3* group. There were no consistent differences of these cell populations across three datasets. For example, high *B7*‐*H3* expressers had significantly lower proportions of resting T cells CD4 memory cells in the TCGA and GSE10358 datasets, but not in the GSE13159 dataset. The opposite pattern was observed for CD8 T cells in the previous two datasets (Figure 6).

To resolve the inconsistent results obtained from CIBERSORT analyses, we further collected immune gene signatures from four studies to compute immune infiltration scores using ssGSEA.^24^, ^25^, ^26^, ^27^ Then, we compared the ssGSEA scores computed for high *B7*‐*H3*‐expressing samples with those in low *B7*‐*H3*‐expressing samples. Significant differences were found between two groups in estimates for a number of immune populations (FDR <0.05), across different gene signature and data sources (Data S2). Table 1 shows the number of differentially enriched cell populations across four gene signature sources in at least 1 dataset. Notably, there was a significant increase in several immunosuppressive cells (i.e., macrophages, neutrophils, dendritic cells, Th17 cells, CD56dim natural killer cells, and monocytes) in patients with high *B7*‐*H3*,while cells executing anti‐tumor reactivity (i.e., activated CD4 T cells, effector memory CD4 T cell, and activated CD8 T cells) were generally under‐represented (Table 1). This indicates that *B7*‐*H3* may play a pro‐tumourigenic immune suppressive role in the tumor microenvironment.

**TABLE 1:** The number of differentially enriched cell populations across four gene signature sources in at least 1 dataset (TCGA microarray, GSE10358, and GSE13159).

Gene Signatures	Angelova	Bindea	Charoentong	Senbabaoglu	Sum
Up‐regulated in high *B7‐H3*					
Macrophage	2	2	2	2	8
Neutrophil	2	2	2	2	8
Dendritic cell	2	2	1	3	8
Type 17 T helper cell	2	2	2	2	8
CD56dim natural killer cell	1	1	2	1	5
Monocytes	1	0	3	0	4
Mast cell	1	0	2	0	3
MDSC	1	0	2	0	3
Regulatory T cell	0	0	2	0	2
Down‐regulated in high *B7‐H3*					
Memory B cell	3	0	3	0	6
B cell	0	1	0	2	3
T helper cell	0	1	0	2	3
Activated CD4 T cell	1	0	1	0	2
Effector memory CD4 T cell	1	0	1	0	2
Activated CD8 T cell	1	0	0	0	1
Natural killer cell	0	0	0	1	1

**FIGURE 7:**
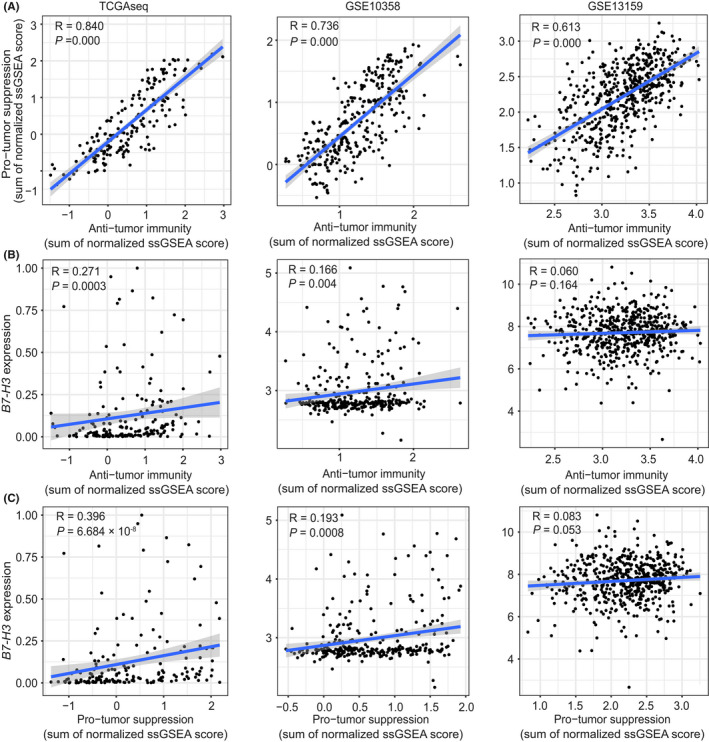
*B7*‐*H3* expression is associated with immune‐suppressive cell populations in AML. (A) Correlation between summarized ssGSEA scores of immune subtypes executing anti‐tumor immunity (ActCD4, ActCD8, TcmCD4, TcmCD8, TemCD4, TemCD8, Th1, Th17, ActDC, CD56briNK, NK, NKT) and cell types executing pro‐tumor, immune suppressive functions (Treg, Th2, CD56dimNK, imDC, TAM, MDSC, Neutrophil, and pDC) across three datasets (TCGA microarray, GSE10358, and GSE13159). (B,C) Correlation between *B7*‐*H3* expression and summarized ssGSEA scores of immune subtypes executing anti‐tumor immunity (B) and cell types executing pro‐tumor, immune suppressive functions (C) across three datasets (TCGA microarray, GSE10358, and GSE13159). Spearman correlations and p values are indicated. The linear models describing the correlations are depicted as blue lines

To evaluate this hypothesis, 20 of 28 immune gene signatures published by Charoentong et al^26^ were classified as “immune‐active” (*n* = 12) and “immune‐suppressive” (*n* = 8) subtype as defined by Jia Q et al.^30^ We observed a strong correlation of the summarized ssGSEA scores between two immune subtypes in all three datasets (Spearman *p* = 0.000, Figure 7A). This is consistent with previous observation in lung cancers, which may reflect a concomitant counter‐activation of immune suppression associated with immune‐activation. Further analyses revealed that *B7*‐*H3* expression was associated with anti‐tumor immunity in two of the three datasets (Figure 7B); however, the association between *B7*‐*H3* expression and pro‐tumor suppression was much more significant across three datasets (Figure 7C). Overall, these results indicate that the role of *B7*‐*H3* in AML may be more inclined to immune suppression.

### 3.8 Correlation between B7‐H3 and other immune checkpoints in AML

**FIGURE 8:**
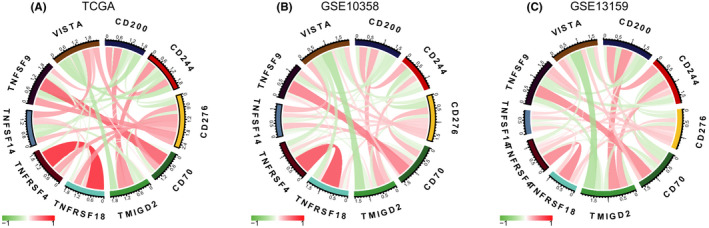
Correlation between *B7*‐*H3* and other immune checkpoints in AML. (A, B, and C) TCGA microarray (A), GSE10358 (B), and GSE13159 (C) datasets

Given that immune checkpoints have been proved to be promising therapeutic target for cancer treatment, we also evaluated the relationship between *B7*‐*H3* and a collection of checkpoint genes describe by De Simone et al.^36^ Results from Spearman correlation analysis across three datasets are given in Data S3. Nine genes were constantly associated with *B7*‐*H3* in all three datasets as shown by Circos plots (Figure 8). Interestingly, four genes from the Tumor Necrosis Factor family‐*TNFRSF4* (*OX40*), *TNFSF9* (*CD137L*), *TNFSF14* (*LIGHT*), and *TNFRSF18* (*GITR*)‐were among the top genes that were positively correlated with *B7*‐*H3* expression. Two other genes that showed significant positive correlations with *B7*‐*H3* were *VISTA* (*B7*‐*H5*) and *CD70*. Notably, both preclinical models and an early clinical trial have shown that blocking *CD70* in combination with hypomethylating agents as a promising and effective therapeutic strategy for AML patients.^37^, ^38^ Genes had constant negative correlations with *B7*‐*H3* were *CD200*, *CD244*, and *TMIGD2*, in which *TMIGD2* was recognized as a receptor for the recently identified B7 family member HHLA2.^39^

Except for VISTA (B7‐H5), no correlations were found between other B7 family checkpoints and *B7*‐*H3*, or significant correlation was only found in one or two datasets for genes including *ICOSLG* (*B7*‐*H2*), *VTCN1* (*B7*‐*H4*), *CD80* (*B7*‐*1*), and *CD86* (*B7*‐*2*) (Data S3). Finally, *PD*‐*1* and *CTLA*‐*4*, two most widely studied immune checkpoints, were found to be positively correlated with *B7*‐*H3* expression in the GSE13159 dataset.

### 3.9 High expression of B7‐H3 was associated with poor outcomes in AML patients

**FIGURE 9:**
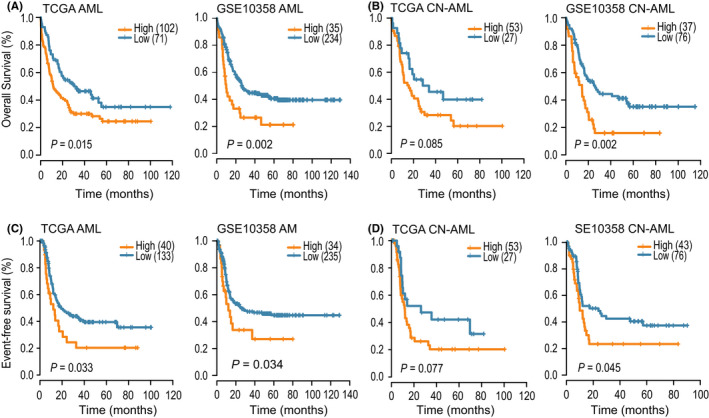
High expression of *B7*‐*H3* was associated with poor outcomes in AML patients. (A,B) OS of the whole cohort (A) and CN‐AML (B) patients in the TCGA and GSE10358 datasets, according to *B7*‐*H3* expression status. (C,D) EFS of the whole cohort (C) and CN‐AML (D) patients in the TCGA and GSE10358 datasets, according to *B7*‐*H3* expression status

Next, we analyzed the relevance of *B7*‐*H3* expression to patient survival in the TCGAseq and GSE10358 datasets. The patients from each dataset were divided into two groups according to *B7*‐*H3* expression and patients’ survival using the maxstat method. Kaplan–Meier analysis demonstrated that high *B7*‐*H3* expression at diagnosis was significantly associated with worse OS and EFS within the whole cohort for both datasets (Figure 9A,C). A similar pattern was observed in the Kaplan–Meier curves of cytogenetically normal (CN)‐AML patients (Figure 9B,D). Importantly, these findings were further validated in two independent CN‐AML cohorts (GSE12417 [U133plus2], *n* = 79; GSE71014, *n* = 104) for OS (Figure S7). Collectively, these data indicated that *B7*‐*H3* is a negative prognostic indicator in AML patients.

## 4 DISCUSSION

Using transcriptional data of cancer cell lines from CCLE and HPA,we demonstrated that *B7*‐*H3* expression was relatively low in hematologic malignancies as compared with solid tumors, while AML cell lines displayed the highest expression levels among hematologic malignancies. Further analyzing over 2000 patient samples encompassing five major hematologic malignancies and normal controls, we found that *B7*‐*H3* expression was highest in AML patients and lowest in ALL. This is consistent with previous data obtained from flow cytometry: fluorescent signals of *B7*‐*H3* were relatively weak in lymphocytes but strong in myeloid leukemia cells.^19^ Also consistent with the previous report was the observation of higher *B7*‐*H3* expression in the M3 and M5 FAB subtypes and in patients with wild‐type CEBPA. Although our results were limited to a narrow view of *B7*‐*H3* expression patterns in AML by focusing only mRNA levels, the strengths of our study include the large sample size and independent validation in different patient cohorts. More importantly, we think that both approaches complement and validate each other and together strongly indicate that *B7*‐*H3* up‐regulation is a common event in AML.

Another study by Hu Y et al,^18^ analyzing both mRNA and protein expression of *B7*‐*H3* in AML, have reported somewhat inconsistent results with Guery T et al.^19^ First, as opposed to Guery T et al,^19^ Hu Y et al^18^ did not find any differences in *B7*‐*H3* expression among different FAB classifications. Second, while Hu Y et al^18^ has reported that *B7*‐*H3*‐positive cases were more likely to have unfavorable karyotypes, no significant association between *B7*‐*H3* and cytogenetic risk was observed by Guery T et al.^19^ Lastly, Hu Y et al^18^ showed that *B7*‐*H3* expression predicts worse outcome in acute leukemia (AL); in contrast, significantly better EFS and in trend a better OS in *B7*‐*H3*‐positive patients was observed by Guery T et al.^19^ Our results on the prognostic relevance are in line with Hu Y et al^18^ showing that high *B7*‐*H3* expression was associated worse clinical outcome. There are several potential explanations for the different findings with respect to the prognostic impact of *B7*‐*H3*. The two previous studies were based on smaller cohort of patients (less than 100) and shorter follow‐up time; moreover, the study by Hu Y et al^18^ comprised both AML and ALL patients and the chemotherapy regimen was not indicated. Our analysis, in contrast, covers four larger patient cohorts (625 in total) with longer follow‐up data and, the prognostic value of *B7*‐*H3* was also assessed in more uniformly treated AML patients with normal cytogenetics. Additionally, quantifying *B7*‐*H3* expression at mRNA or protein levels might affect the prognosis assessment. Indeed, *B7*‐*H3* protein has been demonstrated to exert both co‐inhibitory and co‐stimulatory effect in T‐cell activation,^9^ thereby mediating pro‐ or anti‐tumor activities depending on the immune contexture and tumor types. The inconsistencies might also be caused by differences in age, race, and ethnic group among study cohorts, for which different genetic and environmental factors might affect the elements of cancer immunity in AML. Obviously, additional studies involving both *B7*‐*H3* mRNA and protein expression in significantly larger cohorts are necessary to establish firmly the prognostic value of *B7*‐*H3* in AML.

We also extended our analysis toward the genomic (point mutations and gene copy number gains or losses) and transcriptomic features associated with *B7*‐*H3*, which allowed us to gain further insight into the functional significance of *B7*‐*H3* expression in AML. As for copy number alterations, we found that in high *B7*‐*H3* expressers, several tumor suppressors (*CDKN1B*, *ETV6*, *DUSP16*, and *miR*‐*613*) was deleted, whereas the poor prognosticator *ERG* was amplified, indicating a potential oncogenic role for *B7*‐*H3*. More evidences were provided by genome‐wide *B7*‐*H3*‐associated gene‐expression signatures and following GSEA analysis, whereby several oncogenes and oncogenic gene signatures were significantly enriched in patients with high *B7*‐*H3* expression. Notably, we found two genes implicated in EMT process (*TWIST1* and *MMP7*) was significantly associated with *B7*‐*H3* expression. GSEA analysis also revealed the enrichment of the “apical junction” and “EMT” hallmarks. EMT is a critical step driving tumor invasion and metastasis; these two processes have recently been reported as non‐immunological roles played by *B7*‐*H3* in cancer progression.^40^, ^41^ In U937 AML cell line (M5 subtype), Zhang W et al have shown that *B7*‐*H3* knockdown significantly reduced the migratory rate and invasive capacity of the cell, and also the expression of two EMT regulators (*MMP*‐*2* and *MMP*‐*9*).^42^ Importantly, we have previously demonstrated that dysregulation of EMT genes could be an early event in leukemogenesis and may have profound prognostic implications in AML. Therefore, identifying a possible interaction between *B7*‐*H3* and the EMT program will be of particular interest in future studies.

Previously, it was found that *B7*‐*H3* is preferentially expressed on the monocytic lineage.^19^ This was consistent with the higher *B7*‐*H3* levels their group and ours observed in patients with M5 subtypes. In accordance with these findings, we reported here that, in patients with high *B7*‐*H3* expression, monocytes and macrophages were highly enriched. It is noteworthy that macrophages, which are differentiated from monocytes, turned out to be the top differentially infiltrated cell types (8 comparisons) across different gene signature and data sources. Furthermore, *B7*‐*H3* was found to be positively associated with *CD163* and *CD204*, markers of the immunosuppressive/ pro‐tumorigenic M2 macrophages. We have recently shown that M2 macrophages fractions were increased significantly in AML compared with normal controls and conferred adverse outcome in AML patients. It has also been shown that AML blasts can polarize monocytes to an M2‐like phenotype.^43^ Therefore, it is reasonable to expect that *B7*‐*H3* may be highly expressed on M2 macrophages in AML, and might also participate in M2 macrophages‐mediated pro‐tumor/immune‐suppressive activities. Clearly, future studies will be required to test these hypotheses.

Despite the breakthrough of checkpoint blockade therapy in solid tumors, its progress in the less immunogenic AML has somewhat lagged behind. Nevertheless, recent studies have shown the combination of hypomethylating agents and checkpoint inhibition as an effective strategy in treating AML.^44^ This may be because hypomethylating agents can up‐regulate checkpoint genes such as *PD*‐*1*, *PD*‐*L1*, and *PD*‐*L2* in AML patients, which in turn serve as targets for checkpoint blockade. In our analyses, we found that *B7*‐*H3* was positively associated with a number of checkpoint genes, such as *VISTA* (*B7*‐*H5*), *CD80* (*B7*‐*1*), *CD86* (*B7*‐*2*), and *CD70*, indicating potential synergistic effects between these molecules. Our PPI network analysis also revealed a direct interaction between *B7*‐*H3* and *CD70*, consistent with a previous report that *B7*‐*H3* and *CD70* were highly co‐expressed and up‐regulated in multiple tumor types.^45^ Interestingly, a recent preclinical study has reported that combining decitabine treatment with *CD70* blockade significantly reduced AML LSC frequencies both in vitro and in vivo, with HSCs only marginally affected.^37^ On the other hand, *B7*‐*H3* was reported by Hu Y et al to be preferentially expressed on CD34+ cells^18^; we similarly demonstrated the up‐regulation of *B7*‐*H3* in LSCs as compared to HSCs. We have also shown that *B7*‐*H3* expression might potentially be regulated by DNA methylation. Hence, we deemed that *B7*‐*H3* blockade as a monotherapy, or in combination with hypomethylating agents, might be a promising avenue of therapeutic intervention in AML, which deserves future preclinical and clinical investigations.

In summary, our study confirms and extends the findings of two previous studies for the expression patterns and clinical significances of *B7*‐*H3* in AML. A key advantage of our analyses was the enlarged sample size and independent validations using different datasets. In addition, we uncovered distinct genomic and immunologic features associated with *B7*‐*H3* expression in AML that may lead to better understanding of the molecular mechanisms underlying B7‐H3 dysregulation and to the development of novel therapeutic strategies.

## ETHICS APPROVAL AND CONSENT TO PARTICIPAT

All informed consents were obtained from all patients from respective cohorts. The article was completely based on data from public sources, the ethics of public data has been established already. It was not involving our own data and patients.

## DISCLOSURE STATEMENT

No potential conflicts of interest were disclosed.

## Supporting information

Fig S1‐S7

Data S1

Data S2

Data S3

## Data Availability

The datasets analyzed in this study are available in the following open access repositories: TCGA, http://www.cbiop ortal.org, http://gdac.broad insti tute.org. GEO, https://www.ncbi.nlm.nih.gov/geo/ (GEO accession numbers: GSE13159, GSE63270, GSE30029, GSE10358, GSE12417, GSE71014).

## References

[1] Smyth MJ , Ngiow SF , Ribas A , et al. Combination cancer immunotherapies tailored to the tumour microenvironment. Nat Rev Clin Oncol. 2015;13(3):143‐158. doi:10.1038/nrclinonc.2015.209 26598942

[2] Brahmer JR , Tykodi SS , Chow LQ , et al. Safety and activity of anti‐PD‐L1 antibody in patients with advanced cancer. N Engl J Med. 2012;366(26):2455‐2465. doi:10.1056/NEJMoa1200694 22658128 PMC3563263

[3] Topalian SL , Hodi FS , Brahmer JR , et al. Safety, activity, and immune correlates of anti‐PD‐1 antibody in cancer. N Engl J Med. 2012;366(26):2443‐2454. doi:10.1056/NEJMoa1200690 22658127 PMC3544539

[4] Sharma P , Hu‐Lieskovan S , Wargo JA , et al. Primary, adaptive, and acquired resistance to cancer immunotherapy. Cell. 2017;168(4):707‐723. doi:10.1016/j.cell.2017.01.017 28187290 PMC5391692

[5] Curran EK , Godfrey J , Kline J . Mechanisms of immune tolerance in leukemia and lymphoma. Trends Immunol. 2017;38(7):513‐525. doi:10.1016/j.it.2017.04.004 28511816 PMC6049081

[6] Xu ZJ , Gu Y , Wang CZ , et al. The M2 macrophage marker CD206: a novel prognostic indicator for acute myeloid leukemia. Oncoimmunology. 2020;9(1):1683347. doi:10.1080/2162402x.2019.1683347 32002295 PMC6959428

[7] Davidson‐Moncada J , Viboch E , Church SE , et al. Dissecting the immune landscape of acute myeloid leukemia. Biomedicines. 2018;6(4):110. doi:10.3390/biomedicines6040110 PMC6316310 30477280

[8] Chapoval AI , Ni J , Lau JS , et al. *B7–H3*: a costimulatory molecule for T cell activation and IFN‐gamma production. Nat Immunol. 2001;2:269‐274. doi:10.1038/85339 11224528

[9] Hofmeyer KA , Ray A , Zang X . The contrasting role of B7‐H3. Proc Natl Acad Sci USA. 2008;105(30):10277‐10278. doi:10.1073/pnas.0805458105 18650376 PMC2492485

[10] Suh WK , Gajewska BU , Okada H , et al. The B7 family member *B7–H3* preferentially down‐regulates T helper type 1‐mediated immune responses. Nat Immunol. 2003;4(9):899‐906. doi:10.1038/ni967 12925852

[11] Luo L , Chapoval AI , Flies DB , et al. *B7‐H3* enhances tumor immunity in vivo by costimulating rapid clonal expansion of antigen‐specific CD8+ cytolytic T cells. Journal of immunology 2004;173(9):5445‐5450. doi:10.4049/jimmunol.173.9.5445 15494491

[12] Sun Y , Wang Y , Zhao J , et al. *B7–H3* and B7–H4 expression in non‐small‐cell lung cancer. Lung Cancer. 2006;53(2):143‐151. doi:10.1016/j.lungcan.2006.05.012 16782226

[13] Zang X , Thompson RH , Al‐Ahmadie HA , et al. *B7–H3* and B7x are highly expressed in human prostate cancer and associated with disease spread and poor outcome. Proc Natl Acad Sci USA. 2007;104(49):19458‐19463. doi:10.1073/pnas.0709802104 18042703 PMC2148311

[14] Zang X , Sullivan PS , Soslow RA , et al. Tumor associated endothelial expression of *B7–H3* predicts survival in ovarian carcinomas. Mod Pathol. 2010;23:1104‐1112. doi:10.1038/modpathol.2010.95 20495537 PMC2976590

[15] Yamato I , Sho M , Nomi T , et al. Clinical importance of *B7–H3* expression in human pancreatic cancer. Br J Cancer. 2009;101:1709‐1716. doi:10.1038/sj.bjc.6605375 19844235 PMC2778545

[16] Wu CP , Jiang JT , Tan M , et al. Relationship between co‐stimulatory molecule *B7‐H3* expression and gastric carcinoma histology and prognosis. World J Gastroenterol. 2006;12(3):457‐459. doi:10.3748/wjg.v12.i3.457 16489649 PMC4066068

[17] Sun J , Chen LJ , Zhang GB , et al. Clinical significance and regulation of the costimulatory molecule *B7–H3* in human colorectal carcinoma. Cancer Immunol Immunother. 2010;59(8):1163‐1171. doi:10.1007/s00262-010-0841-1 20333377 PMC11030977

[18] Hu Y , Lv X , Wu Y , et al. Expression of costimulatory molecule *B7–H3* and its prognostic implications in human acute leukemia. Hematology. 2015;20(4):187‐195. doi:10.1179/1607845414y.0000000186 25130683

[19] Guery T , Roumier C , Berthon C , et al. *B7–H3* protein expression in acute myeloid leukemia. Cancer Med. 2015;4(12):1879‐1883. doi:10.1002/cam4.522 26376842 PMC5123710

[20] Newman AM , Liu CL , Green MR . Robust enumeration of cell subsets from tissue expression profiles. Nat Methods. 2015;12(5):453‐457. doi:10.1038/nmeth.3337 25822800 PMC4739640

[21] Barbie DA , Tamayo P , Boehm JS , et al. Systematic RNA interference reveals that oncogenic KRAS‐driven cancers require TBK1. Nature. 2009;462(7269):108‐112. doi:10.1038/nature08460 19847166 PMC2783335

[22] Xu ZJ , Ma JC , Zhou JD , et al. Reduced protocadherin17 expression in leukemia stem cells: the clinical and biological effect in acute myeloid leukemia. J Transl Med. 2019;17(1):102. doi:10.1186/s12967-019-1851-1 30922328 PMC6440111

[23] Tamborero D , Rubio‐Perez C , Muiños F , et al. A pan‐cancer landscape of interactions between solid tumors and infiltrating immune cell populations. Clin Cancer Res. 2018;24(15):3717‐3728. doi:10.1158/1078-0432.ccr-17-3509 29666300

[24] Angelova M , Charoentong P , Hackl H , et al. Characterization of the immunophenotypes and antigenomes of colorectal cancers reveals distinct tumor escape mechanisms and novel targets for immunotherapy. Genome Biol. 2015;16:64. doi:10.1186/s13059-015-0620-6 25853550 PMC4377852

[25] Bindea G , Mlecnik B , Tosolini M , et al. Spatiotemporal dynamics of intratumoral immune cells reveal the immune landscape in human cancer. Immunity. 2013;39(4):782‐795. doi:10.1016/j.immuni.2013.10.003 24138885

[26] Charoentong P , Finotello F , Angelova M , et al. Pan‐cancer immunogenomic analyses reveal genotype‐immunophenotype relationships and predictors of response to checkpoint blockade. Cell Rep. 2017;18(1):248‐262. doi:10.1016/j.celrep.2016.12.019 28052254

[27] Şenbabaoğlu Y , Gejman RS , Winer AG , et al. Tumor immune microenvironment characterization in clear cell renal cell carcinoma identifies prognostic and immunotherapeutically relevant messenger RNA signatures. Genome Biol. 2016;17(1):231. doi:10.1186/s13059-016-1092-z 27855702 PMC5114739

[28] Hänzelmann S , Castelo R , Guinney J . GSVA: gene set variation analysis for microarray and RNA‐seq data. BMC Bioinformatics. 2013;14(1):7. doi:10.1186/1471-2105-14-7 23323831 PMC3618321

[29] Ritchie ME , Phipson B , Wu D , et al. Limma powers differential expression analyses for RNA‐sequencing and microarray studies. Nucleic Acids Res. 2015;43(7):e47. doi:10.1093/nar/gkv007 25605792 PMC4402510

[30] Jia Q , Wu W , Wang Y , et al. Local mutational diversity drives intratumoral immune heterogeneity in non‐small cell lung cancer. Nat Commun. 2018;9(1):5361. doi:10.1038/s41467-018-07767-w 30560866 PMC6299138

[31] Robinson MD , McCarthy DJ , Smyth GK . edgeR: a Bioconductor package for differential expression analysis of digital gene expression data. Bioinformatics. 2010;26(1):139‐140. doi:10.1093/bioinformatics/btp616 19910308 PMC2796818

[32] Mayakonda A , Lin DC , Assenov Y , et al. Maftools: efficient and comprehensive analysis of somatic variants in cancer. Genome Res. 2018;28(11):1747‐1756. doi:10.1101/gr.239244.118. 30341162 PMC6211645

[33] Mermel CH , Schumacher SE , Hill B , et al. GISTIC2.0 facilitates sensitive and confident localization of the targets of focal somatic copy‐number alteration in human cancers. Genome Biol. 2011;12(4):R41. doi:10.1186/gb-2011-12-4-r41 21527027 PMC3218867

[34] Marcucci G , Baldus CD , Ruppert AS , et al. Overexpression of the ETS‐related gene, ERG, predicts a worse outcome in acute myeloid leukemia with normal karyotype: a Cancer and Leukemia Group B study. J Clin Oncol. 2005;23(36):9234‐9242. doi:10.1200/jco.2005.03.6137. 16275934

[35] Dorschner MO , Sybert VP , Weaver M , et al. NF1 microdeletion breakpoints are clustered at flanking repetitive sequences. Hum Mol Genet. 2000;9(1):35‐46. doi:10.1093/hmg/9.1.35. 10587576

[36] De Simone M , Arrigoni A , Rossetti G , et al. Transcriptional landscape of human tissue lymphocytes unveils uniqueness of tumor‐infiltrating T regulatory cells. Immunity. 2016;45(5):1135‐1147. doi:10.1016/j.immuni.2016.10.021. 27851914 PMC5119953

[37] Hinterbrandner M , Kallen NM , Lüthi U , et al. Blocking CD70/CD27 signaling in combination with hypomethylating agents eradicates human CD34+ AML stem and progenitor cells in vitro and in vivo. Blood. 2017;130:2652. doi:10.1182/blood.V130.Suppl_1.2652.2652

[38] Ochsenbein AF , Riether C , Bacher U , et al. Argx‐110 targeting CD70, in combination with azacitidine, shows favorable safety profile and promising anti‐leukemia activity in newly diagnosed AML patients in an ongoing phase 1/2 clinical trial. Blood. 2018;132:2680. doi:10.1182/blood-2018-99-118302

[39] Janakiram M , Chinai JM , Zhao A , et al. HHLA2 and TMIGD2: new immunotherapeutic targets of the B7 and CD28 families. Oncoimmunology. 2015;4:e1026534. doi:10.1080/2162402x.2015.1026534 26405587 PMC4570140

[40] Wang J , Chong KK , Nakamura Y , et al. *B7–H3* associated with tumor progression and epigenetic regulatory activity in cutaneous melanoma. J Invest Dermatol. 2013;133(8):2050‐2058. doi:10.1038/jid.2013.114 23474948 PMC3760237

[41] Wang L , Zhang Q , Chen W , et al. *B7–H3* is overexpressed in patients suffering osteosarcoma and associated with tumor aggressiveness and metastasis. PLoS One. 2013;8(8):e70689. doi:10.1371/journal.pone.0070689 23940627 PMC3734259

[42] Zhang W , Wang J , Wang Y , et al. *B7–H3* silencing by RNAi inhibits tumor progression and enhances chemosensitivity in U937 cells. OncoTargets Ther. 2015;8:1721‐1733. doi:10.2147/ott.s85272 PMC4508088 26203263

[43] Mussai F , De Santo C , Abu‐Dayyeh I , et al. Acute myeloid leukemia creates an arginase‐dependent immunosuppressive microenvironment. Blood. 2013;122(5):749‐758. doi:10.1182/blood-2013-01-480129 23733335 PMC3731930

[44] Boddu P , Kantarjian H , Garcia‐Manero G , et al. The emerging role of immune checkpoint based approaches in AML and MDS. Leuk Lymphoma. 2018;59(4):790‐802. doi:10.1080/10428194.2017.1344905 28679300 PMC5872841

[45] Yang M , Tang X , Zhang Z , et al. Tandem CAR‐T cells targeting CD70 and B7–H3 exhibit potent preclinical activity against multiple solid tumors. Theranostics. 2020;10(17):7622‐7634. doi:10.7150/thno.43991 32685008 PMC7359081

