# Supplementary material for: Integrated analysis reveals distinct molecular, clinical, and immunological features of B7‐H3 in acute myeloid leukemia
Source: Cancer Med. 2021 Sep 25;10(21):7831–46. doi: 10.1002/cam4.4284 (PMC8559480; doi:10.1002/cam4.4284)
Supplement: Supplementary file 1 — Fig S1‐S7 [file CAM4-10-7831-s001.docx]

**Supplementary Information**

**Integrated analysis reveals distinct molecular, clinical and immunological features of *B7-H3* in acute myeloid leukemia**

**This file contains Supplementary Figures (Supplementary Figures S1-S7).**


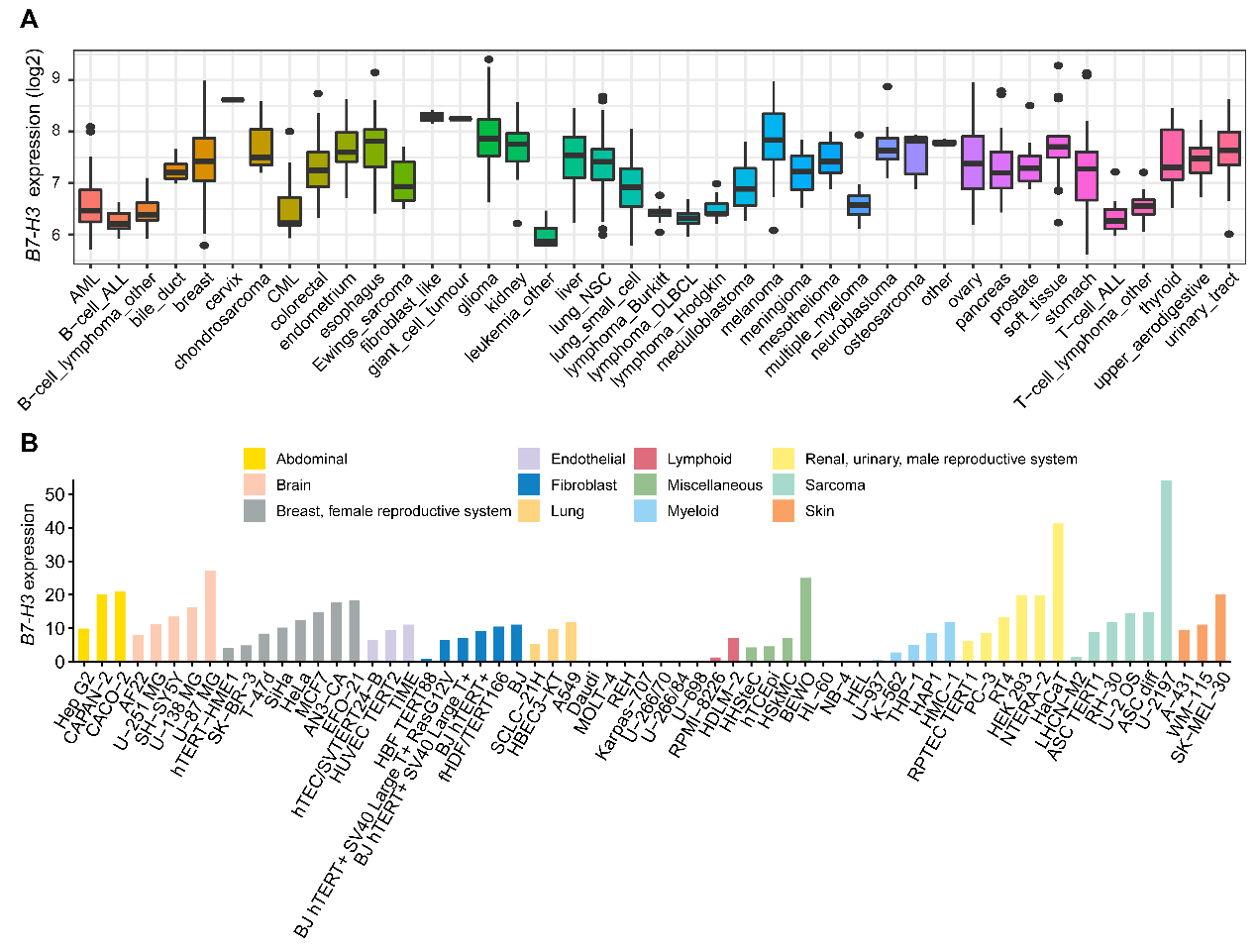


**Supplementary Figure S1. *B7-H3* expression in human cell lines.** **(A)** *B7-H3* mRNA expression levels (RNA microarray data) across cancer cell lines from the Cancer Cell Line Encyclopedia (CCLE) (https://www.broadinstitute.org/ccle). **(B)** *B7-H3* mRNA expression levels (RNA-seq data) in 64 cell lines from The Human Protein Atlas (HPA) (https://www.proteinatlas.org/).


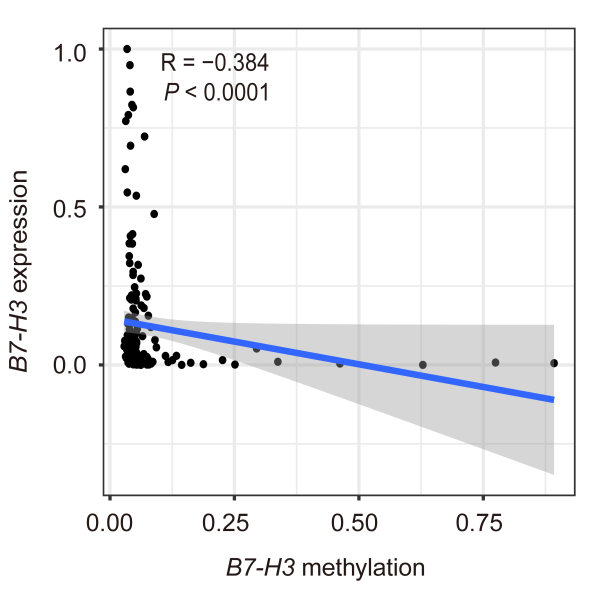


**Supplementary Figure S2. Correlation between *B7-H3* expression and methylation levels in the TCGA dataset.** *B7-H3* expression had significant negative correlation with its methylation level. The Spearman correlation and the p-value are indicated.


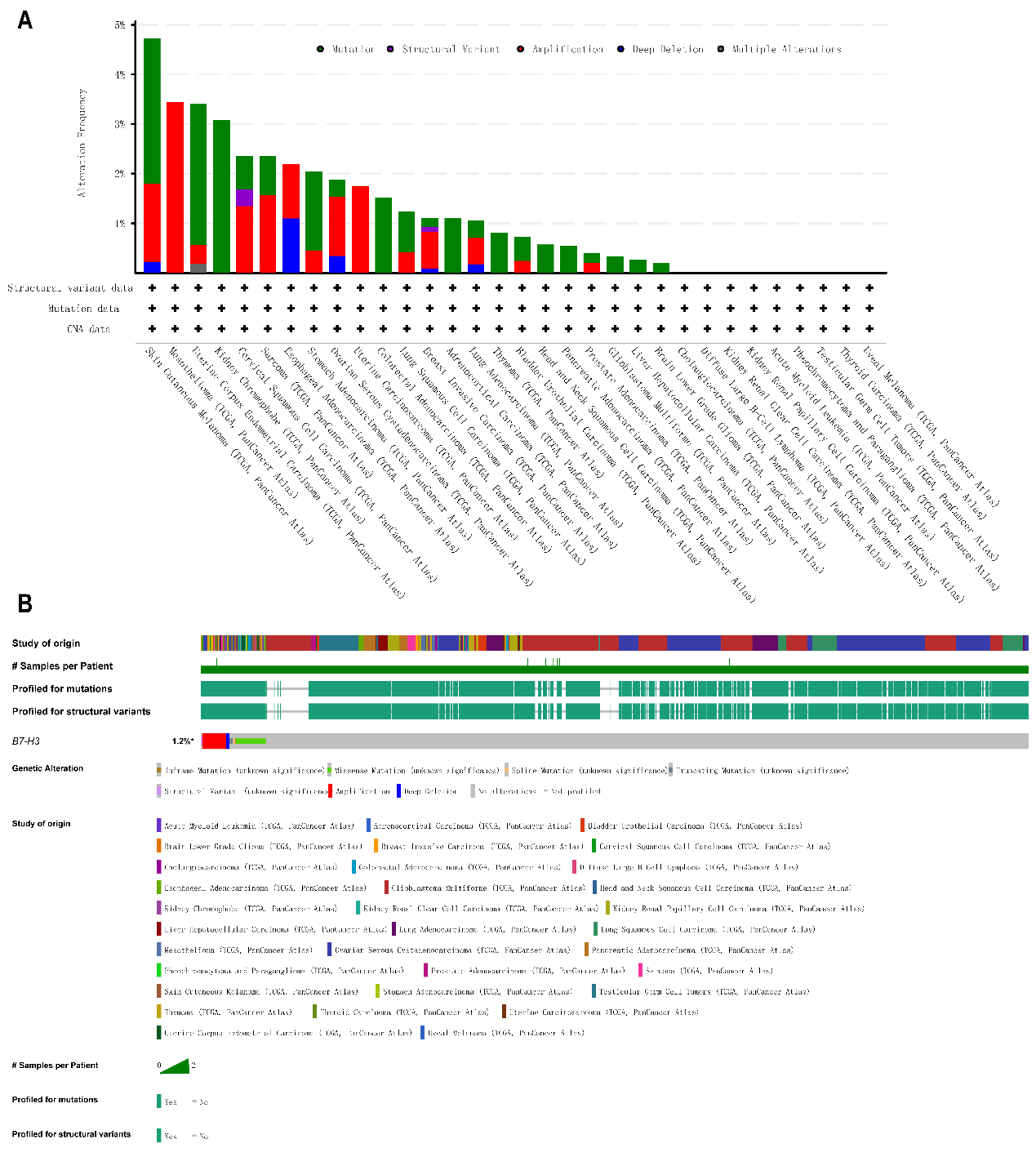


**Supplementary Figure S3.** **The genetic alterations of** ***B7-H3* in pan-cancers. (A)** Genetic alteration frequencies of *B7-H3* across different tumors from TCGA, as assessed by the cbioportal for Cancer Genomics (http://www.cbioportal.org). **(B)** cBioPortal Oncoprint showing the mutation spectrum of *B7-H3* across TCGA pan-cancer studies. Each vertical bar represents a patient. Due to the large size of the oncoplot, only part of it was shown.


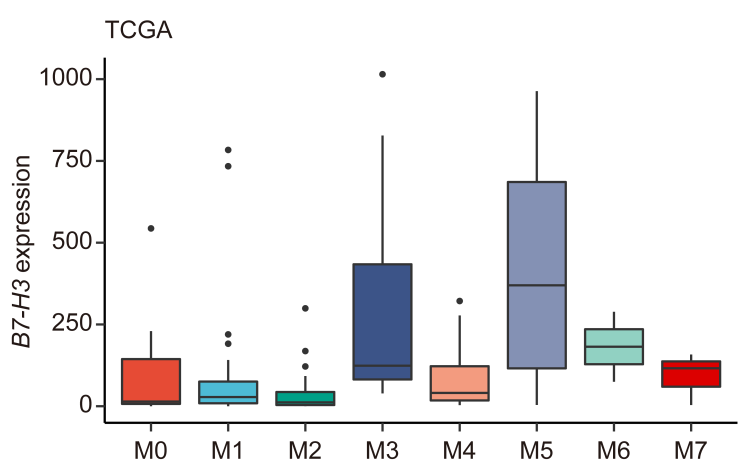


**Supplementary Figure S4. mRNA levels of *B7-H3* in patients with different FAB subtypes from the TCGA cohort.** Data are presented as median and IQR.


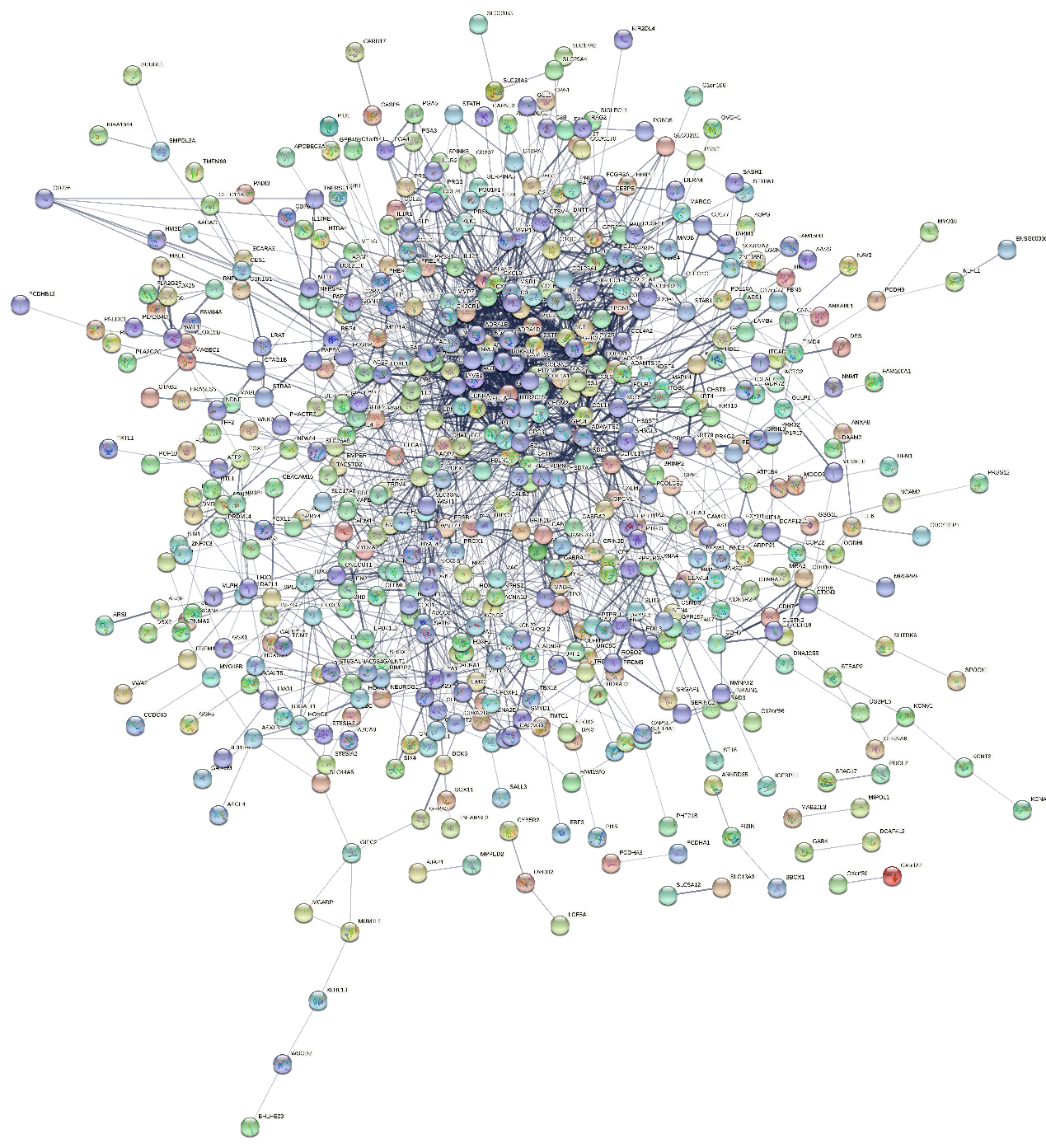


**Supplementary Figure S5. PPI network of differentially expressed genes (DEGs) between patients with high and low *B7-H3* expression by STRING (https ://string-db.org) database.**


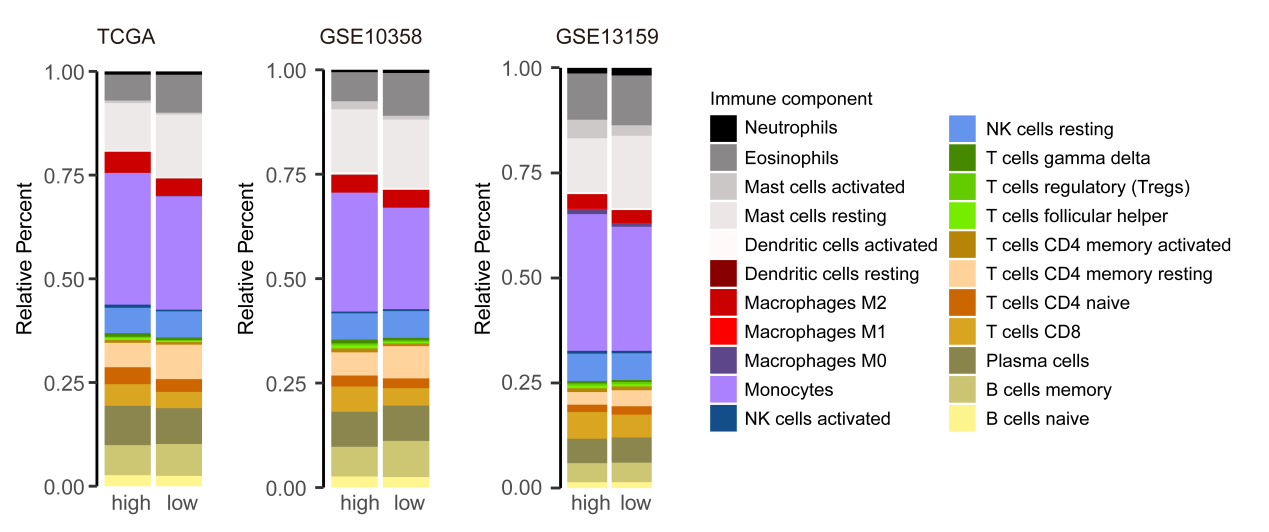


**Supplementary Figure S6. Relative TIL fractions in the BMs of AML patients, as estimated from gene-expression data (TCGA, GSE10358 and GSE13159) using CIBERSORT.** CIBERSORT results are represented as mean TIL fractions across samples for each dataset. Samples with a CIBERSORT p-value ≥  0.05 were removed from this analysis.


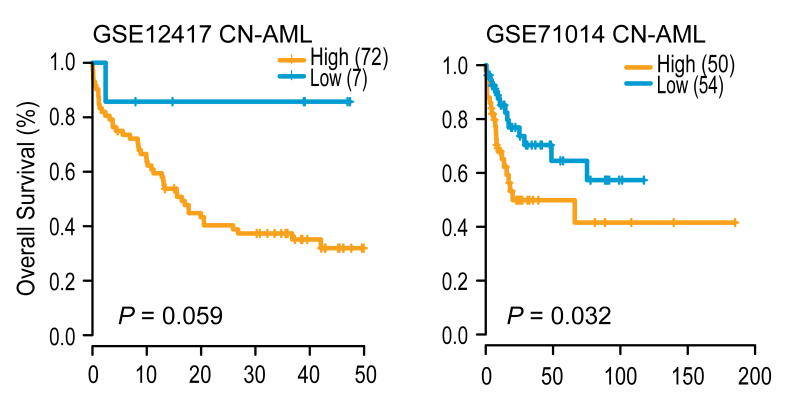


**Supplementary Figure S7. Validation of the prognostic value of *B7-H3* expression in two independent CN-AML cohorts (GSE12417 and GSE71014).**
